# The Role of Bedtime Procrastination, Rumination, Loneliness, and Positive Body Image in Predicting Sleep Quality Among University Students: A Sex-Specific Analysis

**DOI:** 10.31083/AP44142

**Published:** 2025-06-23

**Authors:** Ying Wang, Xiaoyin Wang, Qi Wang, Guoqiu Liu, Chunmei Wu, Ming Hao

**Affiliations:** ^1^School of Public Health and Health Management, Gannan Medical University, University Park, 341000 Ganzhou, Jiangxi, China

**Keywords:** loneliness, positive body image, rumination, sex differences, sleep quality, university students

## Abstract

**Objective:**

This study aimed to analyze the impact of bedtime procrastination, rumination, loneliness, and positive body image on university students’ sleep quality, and to explore potential mediating pathways and sex differences.

**Methods:**

A total of 674 students from a university in southern China were recruited. Assessments of participants’ body measurements were conducted, followed by the completion of a general information questionnaire, Pittsburgh Sleep Quality Index, Bedtime Procrastination Scale, Body Appreciation Scale, Body Image-Acceptance and Action Questionnaire, Ruminative Responses Scale, and University of Loneliness Scale. Stepwise multiple linear regression and mediation models were employed to separately analyze the associations between sleep quality and the aforementioned factors in males and females.

**Results:**

Sex differences in sleep quality were apparent, with women having worse sleep quality than men (*p* < 0.05). In men, bedtime procrastination (β = 0.376, *p* < 0.01), loneliness (β = 0.339, *p* < 0.01), and rumination (β = 0.171, *p* < 0.01) were significant factors in predicting sleep quality. Loneliness played a partial mediating role in predicting poor sleep quality caused by bedtime procrastination, with a mediating effect of 18.95%. In women, bedtime procrastination (β = 0.399, *p* < 0.01), loneliness (β = 0.239, *p* < 0.01), body image flexibility (β = –0.153, *p* < 0.01), and body appreciation (β = –0.103, *p* < 0.05) were significant factors in predicting sleep quality. Loneliness and body appreciation played parallel mediating roles in predicting sleep quality through bedtime procrastination, with mediating effects of 9.24% and 5.19%, respectively.

**Conclusions:**

Sleep quality and bedtime procrastination were worse in women than in men. The sleep quality of female students may be increased by focusing on enhancing positive body image, while for male students, managing rumination and reducing loneliness could be helpful.

## Main Points

- There are sex differences in sleep quality and bedtime procrastination. Women have poorer sleep quality and slightly more bedtime procrastination than men.
- Rumination, loneliness, and bedtime procrastination affected men’s sleep quality, and loneliness had a partial mediating effect in the relationship between bedtime procrastination and sleep quality.
- Bedtime procrastination, loneliness, body appreciation, and body image flexibility affected women’s sleep quality, of which body appreciation and body image flexibility were protective factors. Body appreciation and loneliness played a partial mediating role in the relationship between bedtime procrastination and sleep quality.

## 1. Introduction

Sleep quality is a valuable indicator of physical and psychological health, and happiness. However, according to data from the World Health Organization, 27% of the world’s population have sleep problems [1]. Sleep problems are also a significant concern in China; according to the Chinese Sleep Research Society, more than one-fifth of China’s population have sleep disorders [2]. The Report on National Mental Health Development in China, released in 2021, highlighted the commonality of poor sleep among university students, revealing that 43% of university students believe they are not getting enough sleep [3]. A recent study involving 3423 undergraduate students reported a poor sleep quality prevalence of 43.03% [4], well above the global average. Previous research has shown that there are significant gender differences in sleep quality. Specifically, women generally reported worse sleep quality than men [5]. There are many factors influencing gender differences, including physiological, psychological, and social factors, and it is important to explore the mechanism of these factors to understand the nature of gender differences relating to sleep quality.

University students are a growing group of students in China. Due to the particularity and importance of their life stage, paying attention to their sleep quality and exploring the influencing factors can help solve the challenges they face regarding sleep. Therefore, research on strategies for supporting the healthy growth of university students is paramount.

### 1.1 Relationship between Sleep Quality and Bedtime Procrastination, Rumination, and Loneliness

Bedtime procrastination is a common phenomenon among university students. It is described as an intended postponement of sleep without external circumstances causing delays [6]. In the Netherlands, the proportion of young people who report bedtime procrastination is 53.1% [7]. This unhealthy sleep habit is negatively correlated with sleep duration as well as sleep quality. One factor of concern between bedtime procrastination and sleep quality is rumination. Nolen-Hoeksema defined rumination as a constant focus on one’s negative states without actively addressing real problems [8]. A calm mood is required before going to bed; however, rumination increases an individual’s arousal level, resulting in difficulty in falling asleep. A study conducted in Tokyo on rumination and sleep quality found that rumination predicted a decrease in sleep quality after 3 months [9]. It has also been reported that rumination mediates bedtime procrastination and sleep quality [10]. Regurgitation of this heightened state of cognitive arousal delays the time it takes to fall asleep, leading to decreased sleep quality. Sleep quality is not only closely related to thinking style but also to mood, such as loneliness. For university students, a lack of close relationships and unmet social needs both lead to increased loneliness. A study of freshmen students showed that 75% felt lonely within 2 weeks of starting school [11], and those who self-reported loneliness had poorer subjective sleep quality and more fragmented sleep time [12].

### 1.2 Sleep Quality and Positive Body Image

Positive body image is a popular topic in the field of mental health. It refers to a person’s overall love and respect for their body, and feelings of confidence and happiness with it [13]. Two widely used concepts are body appreciation and body image flexibility. Body image flexibility is the tendency of individuals to be open about their thoughts and feelings regarding their bodies, and act in a manner consistent with their core standards [14]. Body appreciation encompasses a person’s unconditional recognition of and respect for the body, including gratitude and appreciation for physical characteristics, functions, and health [15]. With increasing attention being paid to positive body image, its importance in promoting health-related behaviors has gradually been confirmed. Sleep quality is an important aspect of healthy behavior; however, the relationship between sleep quality and positive body image remains unclear.

Based on this, the purposes of this study were as follows:

(1) To understand the current state of sleep quality among students from a university in southern China and analyze the impact of bedtime procrastination, rumination, loneliness, and positive body image on university students’ sleep quality.

(2) To analyze gender differences in the factors affecting sleep quality and further explore whether positive body image, loneliness, and rumination have a mediating effect on the relationship between bedtime procrastination and sleep quality.

(3) To provide a theoretical basis for clinical practitioners and educators to develop gender-specific, personalized, intervention strategies, ultimately enhancing university students’ sleep quality and overall mental health.

## 2. Material and Methods

### 2.1 Participants

The participants were undergraduate students recruited from a comprehensive university in southern China. Using simple random sampling, questionnaires were distributed in classrooms and student dormitories, and physical measurements were taken by graduate students majoring in public health. The inclusion criteria are as follows: (1) participants must be enrolled university students; (2) age range between 16 and 24 years; (3) voluntary participation with signed informed consent; (4) no serious physical illnesses. The exclusion criteria are: (1) participants who did not sign the informed consent form; (2) missing demographic data; (3) provided inconsistent responses to the questionnaire, such as selecting the same answer for multiple items or showing patterns that indicate a lack of engagement with the questions; (4) age younger than 16 or older than 24; (5) presence of serious physical illnesses. A total of 726 students participated the survey, and after excluding invalid questionnaires, 674 valid responses were obtained, including 316 men and 358 women; their mean age was 18.26 years. The study was conducted between September and November 2023.

### 2.2 Body Measurement

A portable stadiometer (Seca 213, lot number: SM 10000000711205, Manufacturer: Seca gmbh & co. kg, Location: Hamburg, Germany) was used to measure height (0.1 cm accuracy), and a body composition scale (Tanita BC-610, lot number: 5200107, Manufacturer: Tanita Corporation, Location: Tokyo, Japan) was used to measure body weight (0.1 kg accuracy), fat percentage, and muscle mass (0.1 kg accuracy). Height and weight were used to calculate the body mass index (body mass index (BMI); kg/m^2^). Currently, these instruments are used worldwide.

### 2.3 Ruminative Responses Scale

We adopted the Ruminative Responses Scale compiled by Nolen-Hoeksema S and Morrow J [16]. This scale is widely used in Chinese college students. There were 22 questions in total, which were divided into 3 dimensions: symptom rumination, brooding, and reflective pondering. Each item is scored on a four-point scale ranging from 1 (never) to 4 (always). The scores of the 22 items were summed-a higher total score indicated a stronger tendency toward ruminative thinking.

### 2.4 University of Loneliness Scale

The University of Loneliness Scale (ULS-8) was adapted from the scale by Hays and DiMatteo [17]; it contains eight items, each scored on a four-point scale ranging from 1 (never) to 4 (always). The reverse scoring method was adopted for items 3 and 6. The scores of the eight items were summed-a higher score was representative of a higher degree of loneliness.

### 2.5 Pittsburgh Sleep Quality Index

The Pittsburgh Sleep Quality Index (PSQI) was developed by Buysse *et al.* [18]. The 18 questions represent 7 dimensions, including subjective sleep quality, falling asleep time, sleep time, sleep efficiency, sleep disorders, hypnotic drugs, and daytime dysfunction. Each item adopted a four-level frequency scoring method. The scores of the seven dimensions were summed-a higher total score indicated poorer sleep quality, and a sleep quality score greater than 7 is the reference threshold for poor sleep quality [19].

### 2.6 Bedtime Procrastination Scale

The Bedtime Procrastination Scale (BPS) was developed by Kroese *et al*. [6]; it includes nine items in total, and the frequency of occurrence of each situation ranges from 1 (almost never) from 5 (almost always). The reverse scoring method was adopted for items 2, 3, 7, and 9. The total score was the average score of the nine items-a higher score indicated a higher level of bedtime procrastination behavior.

### 2.7 Body Appreciation Scale

The Body Appreciation Scale (BAS-2) was adapted by Swami *et al*. [20]; it includes 10 questions, with responses ranging from 1 (never) to 5 (always) to evaluate the level of agreement. The scores of the 10 items were summed-a higher total score indicated a higher level of appreciation of the individual’s body.

### 2.8 Body Image Acceptance and Action Questionnaire

The Body Image Acceptance and Action Questionnaire (BIAAQ) was developed by Sandoz *et al*. [21]. It involves 12 questions that are assessed on a 7-point Likert scale, ranging from 1 (never correct) to 7 (always correct). Reverse scoring was used for each item. The scores of the 12 items were summed-a higher total score indicated greater body image flexibility.

### 2.9 Statistical Analysis

SPSS (version 19.0; IBM Corp., Armonk, NY, USA) was used for statistical data processing. Detailed descriptive statistics were obtained for the outcomes of each survey. The measurement data were compared using analysis of variance and *t*-tests. Subsequently, a multiple linear regression model was applied to analyze the factors influencing the sleep quality of men and women; the variables were gradually adjusted, and the likelihood ratio test was used to calculate the threshold *p* = 0.2. Ultimately, only the factors that had a significant impact were included in the equation.

Second, we used PROCESS macro (version 4.1, Andrew F. Hayes., Columbus, OH, USA) to conduct a mediation analysis. We explored the relevant mediating effects, and four mediating paths held true. The mediating effect of loneliness on bedtime procrastination and sleep quality were analyzed for both men and women. Moreover, the mediating effect of body appreciation on bedtime procrastination and sleep quality in women, that of rumination on loneliness and sleep quality in men, and that of body appreciation on body image flexibility and sleep quality in women were evaluated. The coefficients of direct and indirect effects were calculated by means of 95% confidence intervals (CIs). All parameters conformed to a normal distribution, and *p* < 0.05 was considered statistically significant. Data are reported as the mean (± standard deviation [SD]) unless otherwise specified.

### 2.10 Sample Size Estimation

Using G*Power software (Version: 3.1.9.7, Manufacturer: Universität Düsseldorf, Location: Düsseldorf, Germany), based on an α-level of 0.05, power (1-β) of 0.90, and five predictors (Bedtime Procrastination Scale score, University of Loneliness Scale score, Ruminative Responses Scale score, Body Image Acceptance and Action Questionnaire score, and Body Appreciation Scale score), the required sample sizes were calculated to detect effect sizes (f^2^) of 0.35 (large), 0.15 (medium), and 0.02 (small), resulting in sample sizes of 53, 116, and 830, respectively. To ensure sufficient statistical power, this study selected a medium effect size (f^2^ = 0.15) and ultimately enrolled 674 participants.

## 3. Results

### 3.1 Gender Differences

The mean age of the respondents was 18.26 ± 0.72 years. Our results revealed that the mean sleep quality score among the respondents was 5.58 ± 3.07, with a prevalence of poor sleep quality of 34.3%. The mean sleep quality score was 5.2 ± 3.0 for men and 5.9 ± 3.1 for women. Sex differences in sleep quality were identified, with the sleep quality of women being worse than that of men (*p* < 0.05). The mean scores for bedtime procrastination behavior were 3.0 ± 0.7 for men and 3.1 ± 0.7 for women (*p* < 0.01) (Table 1).

**Table 1.** **Sample characteristics (n = 674)**.

	Mean ± SD or n (%)		*p*
	Men (n = 316)	Women (n = 358)	
BMI (kg/m^2^)	21.6 ± 3.8	21.3 ± 2.9	0.193
Fat %	16.3 ± 6.5	25.7 ± 5.7	<0.01
Muscle mass (g)	49.8 ± 9.1	37.3 ± 5.1	<0.01
Pittsburgh Sleep Quality Index	5.2 ± 3.0	5.9 ± 3.1	<0.01
Poor sleep quality	94 (29.7)	137 (38.3)	0.023
BPS score	3.0 ± 0.7	3.1 ± 0.7	<0.01
RRS score	42.7 ± 11.9	42.7 ± 11.0	0.917
Symptom rumination	21.9 ± 6.7	22.0 ± 6.2	0.886
Brooding	10.6 ± 3.1	10.6 ± 2.8	0.951
Reflective pondering	10.1 ± 3.1	10.0 ± 3.0	0.525
ULS-8 score	15.2 ± 4.3	15.7 ± 4.3	0.119
BAS-2 score	34.9 ± 7.7	35.7 ± 8.3	0.198
BIAAQ score	59.8 ± 13.0	59.6 ± 12.0	0.830

BMI, body mass index; BPS, Bedtime Procrastination Scale; RRS, Rumination Responses Scale Chinese Version; ULS-8, University of Loneliness Scale; BAS-2, Body Appreciation Scale; BIAAQ, Body Image Acceptance and Action Questionnaire; SD, Standard Deviation.

### 3.2 The Influencing Factors and Mediating Pathways Affecting Sleep Quality in Men

**Fig. 1.**
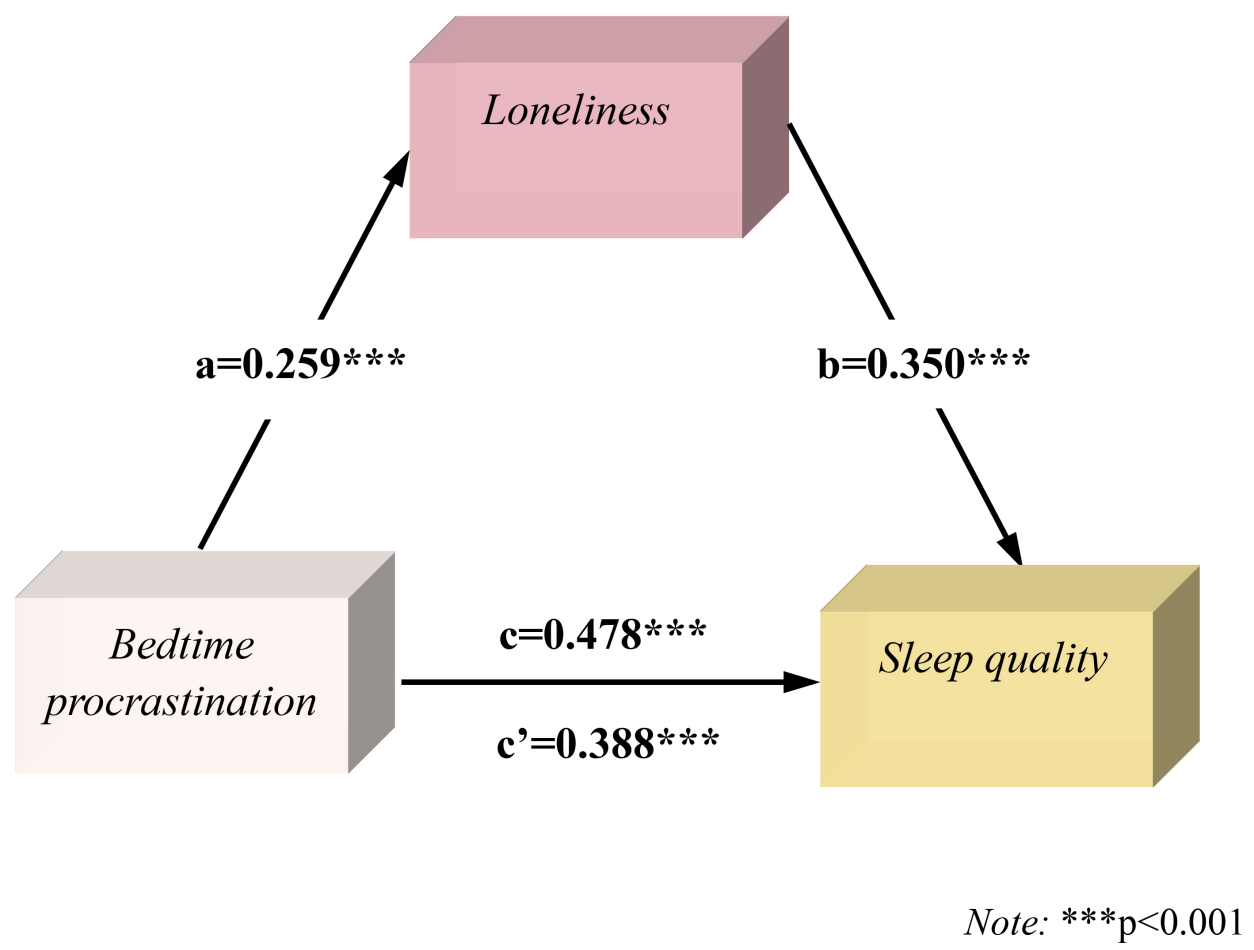
**Mediation model of loneliness between bedtime procrastination and sleep quality in men**.

In men, the bedtime procrastination score (β = 0.376, t = 8.11, *p* < 0.01), loneliness score (β = 0.339, t = 7.32, *p* < 0.01), and ruminative thinking (β = 0.171, t = 3.79, *p* < 0.01) were significant factors predicting sleep quality (Table 2). After loneliness was included in the mediating model between bedtime procrastination and sleep quality (Fig. 1), the bedtime procrastination score had a significant positive predictive effect on the loneliness score (β = 0.259, t = 4.72 *p* < 0.01), and the loneliness score had a significant positive predictive effect on sleep quality (β = 0.350, t = 7.38, *p* < 0.01). The results indicated that the mediating pathway for bedtime procrastination, loneliness, and sleep quality was significant, as was the direct effect of bedtime procrastination on sleep quality (β = 0.388, t = 8.16, *p* < 0.01). Furthermore, the 95% CI [1.120, 1.962] was significant, indicating that loneliness played a partial mediating role in predicting poor sleep quality caused by bedtime procrastination, with a mediating effect of 18.95%.

**Table 2.** **Factors influencing the sleep quality of university students**.

		β	t	VIF	*p*
Men ^‡^					
	BPS	0.376	8.11	1.08	<0.01
	ULS-8	0.339	7.32	1.08	<0.01
	RRS-CV	0.171	3.79	1.02	<0.01
	BMI	–0.079	–1.76	1.01	0.079
Women ^#^					
	BPS	0.399	9.03	1.05	<0.01
	ULS-8	0.239	5.26	1.11	<0.01
	BIAAQ	–0.153	–3.35	1.13	<0.01
	BAS-2	–0.103	–2.19	1.19	<0.05
	BMI	–0.075	–1.72	1.02	0.086

VIF, variance inflation factor; RRS-CV, Rumination Responses Scale Chinese Version. ^‡^ R^2^: 0.38, Root Mean Square Error (RMSE): 2.37. ^#^ R^2^: 0.35, RMSE: 2.52.

### 3.3 The Influencing Factors and Mediating Pathways Affecting Sleep Quality in Women

**Fig. 2.**
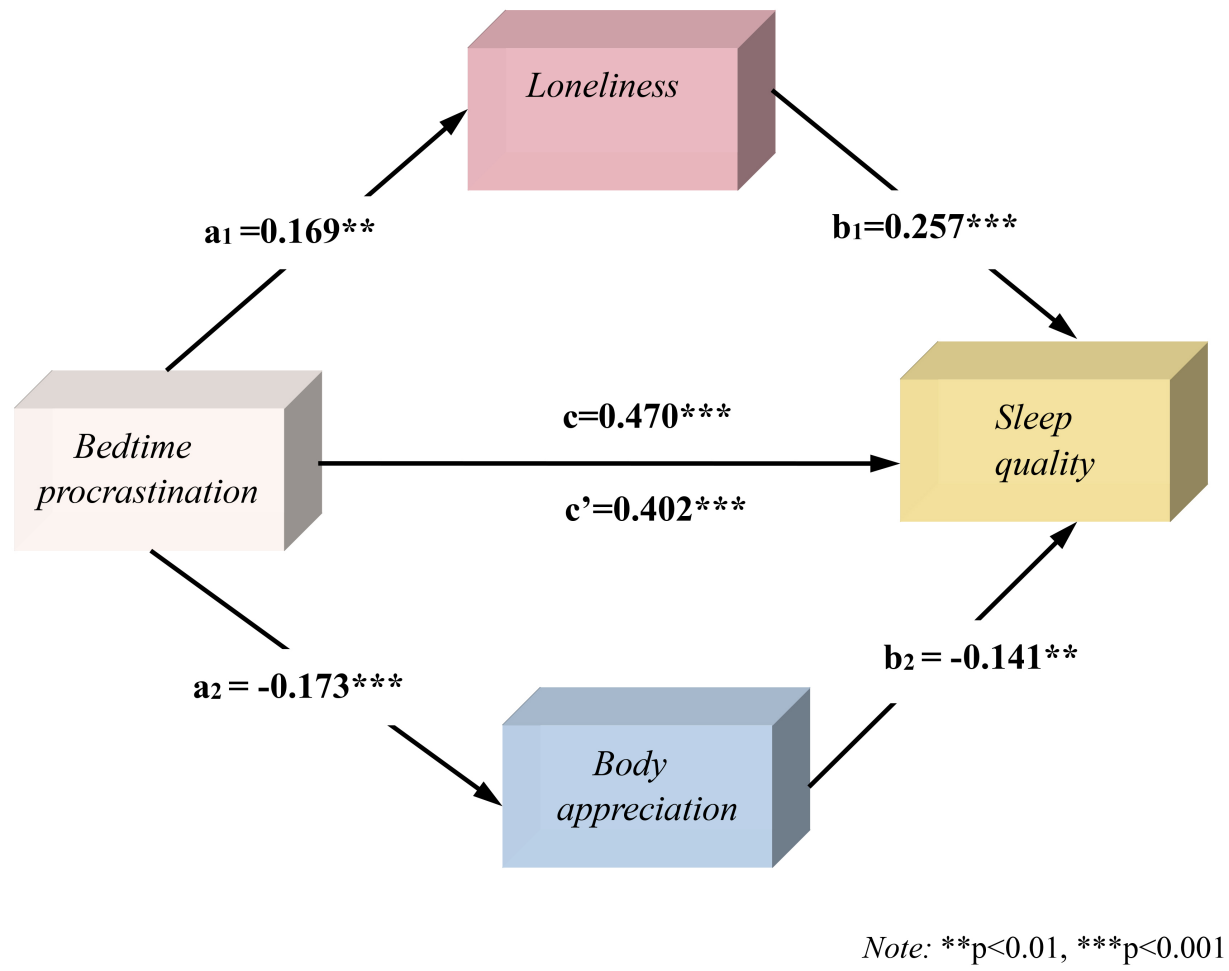
**The mediating role of loneliness and body appreciation between bedtime procrastination and sleep quality in women**.

In women, bedtime procrastination (β = 0.399, t = 9.03, *p* < 0.01), loneliness (β = 0.239, t = 5.26, *p* < 0.01), body image flexibility (β = –0.153, t = –3.35, *p* < 0.01), body appreciation (β = –0.103, t = –2.19, *p* < 0.05) were significant factors predicting sleep quality (Table 2). When evaluating loneliness and body appreciation as mediating variables, the parallel mediating model with bedtime procrastination as the independent variable and sleep quality as the dependent variable (Fig. 2) showed that bedtime procrastination had a significant positive predictive effect on loneliness (β = 0.169, t = 3.24, *p* < 0.01), and loneliness had a significant positive predictive effect on sleep quality (β = 0.257, t = 5.60, *p* < 0.001). This indicated that the mediating pathway of bedtime procrastination, loneliness, and sleep quality was significant, as was the direct effect of bedtime procrastination on sleep quality (β = 0.402, t = 8.98, *p* <0.001). The 95% CI [1.331, 2.077] was significant; thus, we concluded that loneliness plays a partial mediating role in predicting sleep quality caused by bedtime procrastination in women, with a mediating effect of 9.24%. Bedtime procrastination had a significant negative predictive effect on body appreciation (β = –0.173, t = –3.24, *p* < 0.001), and body appreciation had a negative predictive effect on sleep quality (β = –0.141, t = –3.07, *p* < 0.01), indicating a significant mediating pathway between bedtime procrastination, body appreciation, and sleep quality. Moreover, the 95% CI [1.602, 2.381] was significant, indicating that body appreciation played a partial mediating role in predicting sleep quality affected by bedtimeprocrastination, with a mediating effect of 5.19%. Therefore, loneliness and body appreciation played parallel mediating roles in predicting sleep quality through bedtime procrastination.

**Fig. 3.**
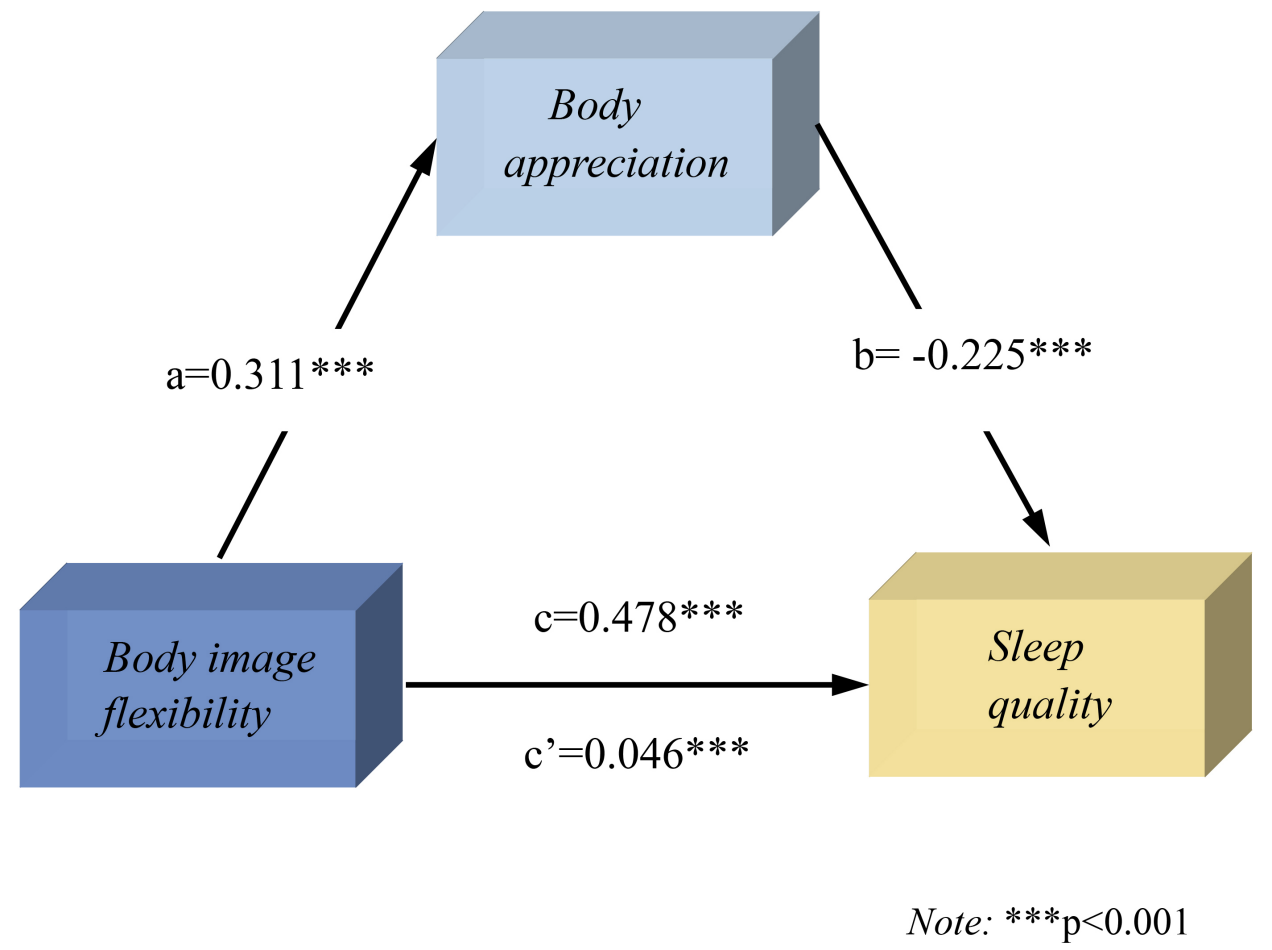
**Mediation model of body appreciation between body image flexibility and sleep quality in women**.

After body appreciation was included in the mediation model between body image flexibility and sleep quality (Fig. 3), body image flexibility had a positive predictive effect on body appreciation (β = 0.311, t = 6.18, *p* < 0.001), and body appreciation had a negative predictive effect on sleep quality (β = –0.225, t = –4.26, *p* < 0.001). This shows that the mediating pathways of body image flexibility, body appreciation, and sleep quality are significant, as was the direct effect of body image flexibility on sleep quality. Additionally, the 95% CI [–0.073, –0.020] was significant, indicating that body appreciation played a partial mediating role in predicting body flexibility on sleep quality, with a mediating effect of 14.62%.

### 3.4 Rumination Partially Mediates the Prediction of Loneliness and Sleep Quality

**Fig. 4.**
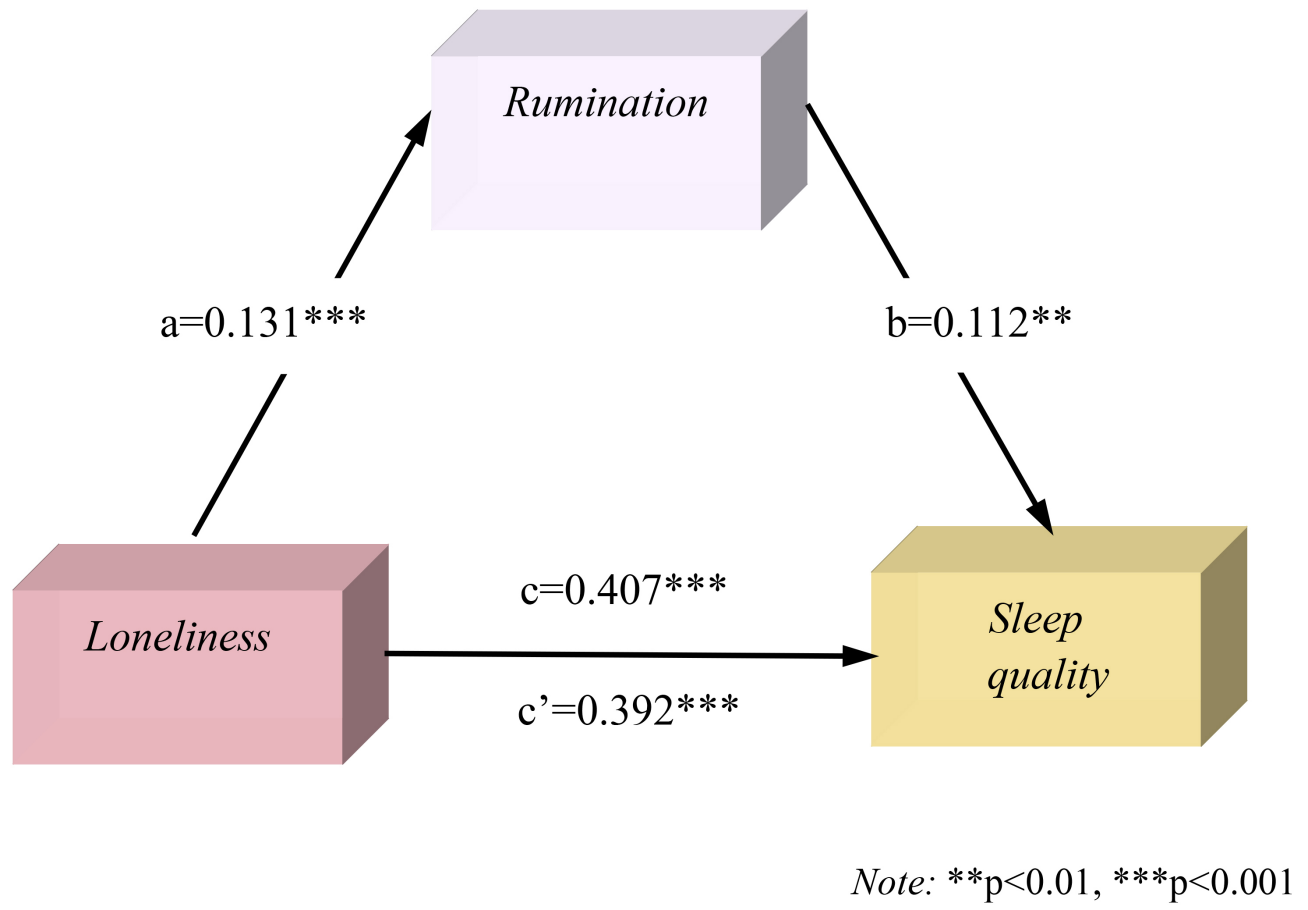
**Mediation model of rumination between loneliness and sleep quality**.

For all participants, when rumination was included in the mediation model between loneliness and sleep quality (Fig. 4), loneliness had a positive predictive effect on rumination (β = 0.131, t = 3.42, *p* <0.001), and rumination positively predicted sleep quality (β = 0.112, t = 3.17, *p* < 0.01). This showed that the mediating pathways of loneliness, rumination, and sleep quality were significant, as was the direct effect of loneliness on sleep quality. Furthermore, the 95% CI [0.228, 0.326] was significant, indicating that rumination played a partial mediating role in predicting loneliness and sleep quality, with a mediating effect of 3.60%.

## 4. Discussion

Our results revealed that the mean sleep quality score among university students was (5.58 ± 3.07), with 34.3% reporting poor sleep quality. In comparison, previous studies reported mean sleep quality scores of 4.9 ± 2.4, with a prevalence of 33.8%, among Taiwanese college students [22], and 4.51 ± 2.52, with a prevalence of 31%, among Chinese students at Jilin University [23]. However, the prevalence of poor sleep quality was notably higher among university students in Hong Kong, reaching 55.8% [24]. In our study sample, the prevalence of poor sleep quality was slightly higher than that of university students from Jilin and Taiwan. This suggests that, despite cultural differences among university students from different regions, they face similarly serious sleep quality issues. However, universities in southern China, such as the one studied herein, may experience slightly worse sleep quality due to more intense academic pressures and a more stressful learning environment. Additionally, environmental factors in southern cities, such as urban noise and air pollution, may also contribute to the poorer sleep quality. In contrast, the difference in sleep quality between our sample and university students in Hong Kong was more pronounced. This may be attributed to the higher academic pressure, lower life satisfaction, and poorer sleep hygiene habits among students in Hong Kong, which make it difficult for them to achieve high-quality sleep [25].

Additionally, this study found a noteworthy difference in sleep quality between men and women. The average sleep quality score of women was 5.9 points, while that of men was 5.2 points. This finding is similar to that of a previous study [26], which reported lower sleep quality in women relative to that in men. Sex was identified as a risk factor for insomnia at the International Conference on the Scientific State of Chronic Insomnia in Adults in 2005 [27]. Objectively, the desynchrony between sleep circadian rhythms and sleep behavior in women may be a contributing factor, potentially related to the unique hormonal changes and effects of ovarian steroid hormones during female puberty [28]. Subjectively, women report more mood changes, anxiety, and depressive symptoms, and these psychological issues affect their sleep more so than they do for men [29].

In addition, this study found a significant sex-based difference in bedtime procrastination, with the bedtime procrastination scores of women and men being 3.1 and 3.0 points, respectively. Bedtime procrastination is closely related to self-regulation ability [7] and is common among university students. Moreover, sleep is generally considered acceptable, whereas procrastination is considered frustrating and is voluntarily performed. Therefore, bedtime procrastination is not an aversion to sleep but an unwillingness to give up other interesting activities or abandon other tasks. The author believes that the reason for this sex difference is that women face more distractions before falling asleep, such as smartphone addiction [30]. With the rapid development of electronic devices and the entertainment industry, losing the sense of time is easy when people unconsciously consume internet content. A study conducted in 2023 in Spain showed that the use of smartphones can indirectly affect sleep quality through sleep procrastination and that the use of smartphones is higher among women [31]. This may be related to the tendency of women to use social networks for satisfaction and social support.

The results of the current study revealed that, in university students, a lower degree of loneliness was significantly associated with higher sleep quality. In addition, loneliness played a partial mediating role in the relationship between bedtime procrastination and sleep quality. The mediating effect of loneliness was 18.9% in men and 9.24% in women, and higher levels of bedtime procrastination were associated with higher levels of loneliness. Loneliness is related to events, social relationships, and attachment behaviors. As the time taken to fall asleep is delayed, thoughts become complicated, moods become depressed, and the experience of loneliness increases. People experiencing loneliness reported more sources of sleep disturbances, such as feeling too cold and nightmares, which are more likely to trigger disrupted sleep [32]. These findings might explain the role of loneliness in bedtime procrastination and sleep quality identified in the present study.

This study further found that rumination had a partial mediating effect on loneliness and sleep quality in all the participants, confirming the hypothesis of Matthews *et al*. [33]. When individuals experience high levels of negative rumination after feeling lonely, excessive brain activity impairs the ability to maintain a calm mood before falling asleep, thereby affecting sleep quality. Furthermore, rumination was identified a predictor of sleep quality in men but not in women. Rumination was not related to bedtime procrastination, and rumination scores did not show sex differences. When sex differences are considered in relation to rumination and sleep quality, they may be explained in terms of social roles and stress. Considering men are often given more responsibility by society, including but not limited to expectations regarding professional or academic success, they may be more inclined to ruminate at night to deal with these pressures, which can affect sleep quality. Similarly, women are more inclined to relax at night when they are under stress and take measures, such as social support and emotional release, to relieve stress [34]. Therefore, we considered the existence of other psychological processes affecting women’s sleep quality; however, this requires further investigation.

This study also found that higher body appreciation and body image flexibility predicted better sleep quality. Body appreciation played a partial mediating role in the relationship between body image flexibility and sleep quality. Furthermore, in women, loneliness acted as a parallel mediator in the relationship between bedtime procrastination and sleep quality. This finding is similar to that of a previous study on a related topic [35]. Body appreciation and body image flexibility are mutually reinforced. Body appreciation is a healthy psychological feeling with a protective effect on sleep quality. Improved sleep quality can reduce fatigue, improve one’s appearance, and enhance body appreciation [36]. If a woman’s body image flexibility is poor, they may engage in self-reflection before going to bed, which affects their sleep quality. With a more flexible body image, a woman may be aware of her thoughts and feelings concerning her body without letting them become obsessions that affect her. The results of this study confirmed the protective role of positive body image on sleep quality, thus supporting the positive body image theory. This indicates that an individual’s positive attitude toward their own body not only contributes to improved self-esteem and mental health but also to enhanced sleep quality. However, research on the association between positive body image and sleep is limited. Nonetheless, this preliminary evidence may serve as a reference for future research.

This study had some limitations. First, as this was a cross-sectional study, we could not make causal inferences. Second, we emphasizes the importance of bedtime procrastination, rumination, loneliness, and positive body image, but it does not encompass all factors related to sleep quality, which focused only on psychological factors that affect sleep quality. Future studies may consider further exploring gender differences by conducting studies with a larger sample size and using different measurement methods to examine the potential impact of body appreciation in male university students. In future, well-designed longitudinal studies that address the challenges associated with sleep health by focusing on both subjective and objective psychological factors that affect sleep quality would be advantageous.

## 5. Conclusions

Female university students sleep worse than their male counterparts. High levels of rumination, loneliness, and bedtime procrastination predict poorer sleep quality, while positive body image can act as a protective factor. Improving sleep quality in female students may benefit from focusing on enhancing positive body image, while for male students, managing rumination and reducing loneliness could be helpful.

## Data Availability

The datasets used and analyzed in the current study are available from the corresponding author upon reasonable request.
